# Case Report: Neonatal-onset chylomicron retention disease presenting as isolated failure to thrive with compound heterozygous *SAR1B* variants: the value of early genetic testing and challenges of long-term management

**DOI:** 10.3389/fped.2026.1684900

**Published:** 2026-02-12

**Authors:** Bing Liu, Chunlei Zhang

**Affiliations:** Department of Neonatology, Weifang Maternal and Child Health Hospital, Weifang, China

**Keywords:** adherence, chylomicron retention disease, dietary intervention, failure to thrive, genetic testing, *SAR1B*

## Abstract

Chylomicron retention disease (CMRD) is a rare autosomal recessive disorder caused by pathogenic variants of *SAR1B*, in which defective intestinal chylomicron secretion leads to fat malabsorption, hypocholesterolemia, and failure to thrive in infancy. Its diagnosis is typically challenging because of its rarity, nonspecific early symptoms, and overlap with other metabolic and malabsorptive disorders. The present case is notable for its neonatal onset with isolated failure to gain weight and the absence of persistent diarrhea or steatorrhea, complicating early clinical suspicion. Initial metabolic screening revealed overlapping abnormalities with urea cycle disorders and fatty acid oxidation defects, underscoring diagnostic complexity. A definitive diagnosis was achieved through the identification of compound heterozygous likely pathogenic *SAR1B* variants, c.258G > A (p.Trp86Ter) and c.442C > T (p.Arg148Ter), thereby expanding the known phenotypic and genotypic spectrum of CMRD. The patient exhibited marked clinical and biochemical improvements following timely intervention with a low-fat, medium-chain triglyceride(MCT)-enriched diet and fat-soluble vitamin supplementation. However, subsequent follow-up revealed suboptimal adherence to the dietary regimen, leading to the emergence of typical steatorrhea, persistent growth failure, and neurodevelopmental delay, highlighting the critical and sustained role of strict nutritional management. This case highlights the importance of heightened clinical vigilance, timely genetic testing, and the necessity of ensuring long-term treatment adherence in infants presenting with unexplained growth failure and subtle hepatic abnormalities even when classic symptoms are absent. Taken together, this study provides valuable insights into the diagnostic challenges, therapeutic pitfalls, and management strategies of CMRD, emphasizing the need for multidisciplinary collaboration, enhanced awareness among clinicians, and further research to elucidate genotype-phenotype correlations and optimize patient care.

## 1 Introduction

Chylomicron retention disease (CMRD), also known as Anderson's disease, is a rare autosomal recessive disorder characterized by the impaired intestinal secretion of chylomicrons owing to pathogenic variants of the *SAR1B* (1, 2). The *SAR1B* encodes a key component of the COPII vesicular transport machinery required for exporting chylomicrons from enterocytes to the lymphatic system (3, 4). The global prevalence of CMRD is estimated to be exceedingly low, with fewer than 100 cases documented in the literature to date, with most cases occurring in populations with a high degree of consanguinity (2, 4). The disease typically presents in early infancy with failure to thrive, chronic diarrhea, steatorrhea, vomiting, and abdominal distension, often accompanied by severe malnutrition. Laboratory analyses frequently reveal hypocholesterolemia, low apolipoprotein B-containing lipoproteins, deficiencies in fat-soluble vitamins, and evidence of hepatic dysfunction, including elevated transaminases (1, 2, 5).

Diagnosing CMRD is particularly challenging because of its rarity, nonspecific early symptoms, and overlap with other causes of malnutrition, malabsorption, and inherited metabolic disorders. Initial presentations of poor weight gain and vomiting are frequently misattributed to more common neonatal issues, delaying appropriate investigation and management. Furthermore, laboratory findings such as hypocholesterolemia and fat-soluble vitamin deficiencies may be mistaken for secondary consequences of malnutrition rather than primary defects in lipid metabolism. Moreover, CMRD must be differentiated from other congenital disorders of lipid absorption, including abetalipoproteinemia and homozygous familial hypobetalipoproteinemia, as well as other inborn errors of metabolism with similar features (6, 7). Therefore, definitive diagnosis increasingly relies on genetic sequencing to identify pathogenic variants in *SAR1B*, often necessitating multidisciplinary collaboration and advanced biochemical and molecular diagnostics (2, 8).

In this study, we present the case of a male infant with neonatal onset of isolated failure to gain weight, without persistent diarrhea or steatorrhea. This case report contributes to the limited international literature on CMRD, particularly for early infancy with a non-classical presentation and rare *SAR1B* genotypes. Moreover, it emphasizes the need for heightened clinical suspicion, multidisciplinary assessment, and the pivotal role of genetic analysis in the diagnosis of rare lipid metabolism disorders. The favorable outcome achieved through appropriate dietary modification further reinforces the clinical significance of early identification and intervention, while the episode of disease progression associated with suboptimal patient adherence highlights the profound impact of long-term nutritional management on clinical outcomes, offering valuable guidance for clinicians encountering similar diagnostic dilemmas (1, 2, 9).

## 2 Case presentation

### 2.1 Patient information

A male infant aged 2 months and 8 days was admitted to the Weifang Maternal and Child Health Hospital in June 2025 owing to a failure to gain weight since birth. He was delivered vaginally at 39 weeks and 3 days of gestation, with a birth weight of 3,420 g. The perinatal history was notable for birth asphyxia requiring resuscitation. Apgar scores were 5, 9, and 10 at 1, 5, and 10 min, respectively. Meconium-stained amniotic fluid and a nuchal cord were noted at delivery, and the placenta appeared normal. The neonate was hospitalized in the neonatal unit for one week after birth. Following discharge, he was exclusively breastfed, with feeding every 2 h and each session lasting approximately 30 min. The infant occasionally vomited and had loose stools with milk curd but had normal urination. At 42 days of age, clinical examination revealed signs of malnutrition (weight stagnation, reduced subcutaneous fat). Given the primary concern of inadequate caloric intake from exclusive breastfeeding, a preliminary diagnosis of “failure to thrive due to probable undernutrition” was considered. Therefore, mixed feeding was recommended, involving supplementation of breast milk with a standard infant formula, aiming to increase overall caloric and protein intake. However, the family did not adhere to this recommendation and continued exclusive breastfeeding. At 2 months and 8 days of age, the infant's weight remained unchanged at 3,400 g. No family history of consanguinity or genetic disorders was noted, and the parents and older sister were asymptomatic.

### 2.2 Clinical findings

Upon physical examination at admission, the infant's weight was 3,400 g (below the 3rd percentile, WHO growth charts), head circumference was 36.6 cm (3rd percentile), and length was 55.2 cm (10th percentile). The patient was alert but presented with loose skin and reduced subcutaneous fat. Respiratory effort was stable, and heart sounds were strong and regular without murmurs. The abdomen was distended but soft, with no hepatosplenomegaly; normal bowel sounds were present. Neurological examination results were unremarkable, and the muscle tone in all limbs was normal.

Upon admission, prior to the confirmation of CMRD and the initiation of disease-specific therapy, a trial of increased caloric intake was implemented to rule out simple undernutrition. The infant was fed with a standard infant formula providing approximately 105 kcal/kg/day (equivalent to approximately 160 mL/kg/day). Despite this adequate nutritional support, which exceeds typical maintenance requirements, the patient continued to demonstrate poor weight gain and static growth parameters. This lack of response to adequate caloric provision pointed towards a primary malabsorptive defect rather than insufficient intake.

Laboratory investigations revealed elevated liver enzymes: alanine aminotransferase (ALT) at 272 U/L (normal: 8–71 U/L) and aspartate aminotransferase (AST) at 242 U/L (normal: 21–80 U/L), along with hypoalbuminemia with albumin at 25.2 g/L (normal: 35–50 g/L), hyperbilirubinemia with total bilirubin at 36.5 μmol/L (normal: 3–24 μmol/L) and direct bilirubin at 9.4 μmol/L (normal: 0–6 μmol/L), hyperammonemia with blood ammonia at 82.3 μmol/L (normal: 15–45 μmol/L). Lipid analysis revealed hypocholesterolemia, with total cholesterol at 1.76 mmol/L (normal: 3.60–6.5 mmol/L), triglycerides at 0.75 mmol/L (normal: 0.4–1.71 mmol/L), high density lipoprotein cholesterol at 0.61 mmol/L (normal: 0.9–2.0 mmol/L), and low density lipoprotein cholesterol at 0.66 mmol/L (normal: 2.0–3.1 mmol/L). Coagulation studies showed a prolonged activated partial thromboplastin time (41.2 s; normal: 24.0–35.0 s), decreased fibrinogen (1.0 g/L; normal: 2.0–4.0 g/L), and mildly elevated international normalized ratio at 1.21 (normal: 0.8–1.2). Thyroid function tests indicated low free thyroxine (7.5 pmol/L; normal: 11.5–28.3 pmol/L) and normal thyroid stimulating hormone (4.13 μIU/mL) levels. Blood urea nitrogen was normal (1.9 mmol/L), creatinine was normal (22 μmol/L), glucose was normal (4.4 mmol/L), and arterial blood gas were within normal limits. Serological tests for TORCH infections, EBV-DNA, and CMV-DNA were negative. Complete blood count and peripheral smear results were within normal limits. Urinalysis showed no ketones. Notably, apolipoprotein B and other fat-soluble vitamin levels (A, E, K) were not measured at presentation due to local laboratory constraints. Follow-up serum 25-hydroxyvitamin D level after diagnosis and supplementation was 56.1 ng/mL. Ophthalmological slit-lamp examination revealed no retinopathy or cataracts. Echocardiography and abdominal ultrasonography findings were unremarkable. Brain MRI revealed age-appropriate myelination. A few non-specific, punctate short T1 signals were noted in the left parietal cortex; their clinical significance is uncertain, and they may represent incidental findings or perinatal asphyxia,unrelated to the primary metabolic diagnosis.

Tandem mass spectrometry of blood revealed elevated citrulline (46 µmol/L) and abnormal amino acid ratios suggestive of argininosuccinate lyase deficiency, type I citrullinemia, or possible hyperornithinemia-hyperammonemia-homocitrullinuria syndrome to be excluded. Urine organic acid analysis revealed increased levels of dicarboxylic acids and 3-hydroxybutyrate, raising the suspicion of fatty acid oxidation disorders or secondary metabolic changes.

Genetic testing was performed on the proband and both parents using trio-based whole-exome sequencing (WES) (see Supplementary File 1) Genomic DNA was captured using the IDT xGen® Exome Research Panel v2.0 and sequenced on an Illumina platform. Data analysis was conducted following standard bioinformatics pipelines. Genetic testing identified compound heterozygous variants in *SAR1B* (NM_016103.4): c.442C > T (p.Arg148Ter) inherited from the father and c.258G > A (p.Trp86Ter) inherited from the mother. The c.258G > A variant is novel and has not been previously reported in the literature or population databases (dbSNP and gnomAD). Both variants were classified as likely pathogenic according to the ACMG criteria. The c.442C > T variant was rated as PVS1_Strong (predicted loss-of-function in a gene where LOF is a known mechanism), PM2_Supporting (absent or very low frequency in population databases), and PM3 (observed in trans with another pathogenic variant). The c.258G > A variant was rated as PVS1 (predicted loss-of-function) and PM2_Supporting.Diagnostic Assessment.

Based on clinical presentation, laboratory findings, metabolic profiles, and genetic analysis, the patient was diagnosed with CMRD. The diagnosis was supported by the presence of persistent failure to thrive despite adequate caloric intake, hypocholesterolemia, abnormal liver function test results, and the identification of pathogenic *SAR1B* variants. At admission, the patient's stool was described as loose with milk curd, but not overtly fatty. However, upon follow-up after diagnosis, typical steatorrhea (5–7 pale, bulky, foul-smelling stools per day) emerged and persisted.

### 2.3 Therapeutic intervention

The patient was initially managed with a low-fat diet supplemented with an MCT-enriched formula (containing 98% MCT), along with oral supplementation with fat-soluble vitamins (A, D, E, and K1) and essential fatty acids. This nutritional approach aimed to reduce fat malabsorption and improve the nutritional status.

### 2.4 Follow-up and outcomes

Initial Response: Initial response to the prescribed low-fat, high-MCT formula (containing 98% MCT) and fat-soluble vitamin supplementation was encouraging. Within the first two weeks under hospital supervision, vomiting resolved and the infant gained 400 g.

**Figure 1:**
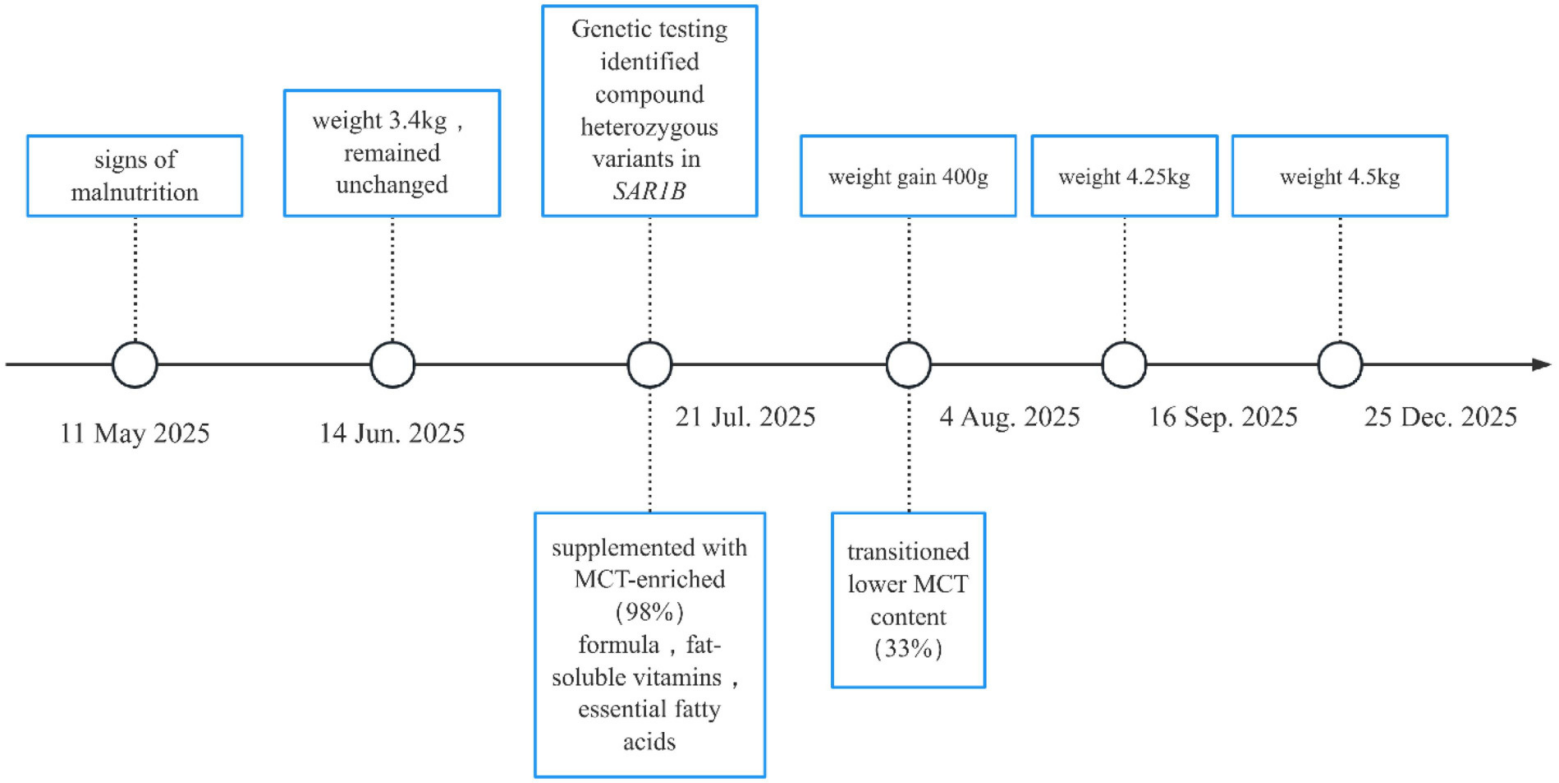
Timeline of key events in the case report.

Long-term Follow-up and Challenges: However, long-term adherence to the recommended dietary regimen proved challenging for the family. Due to the significant economic burden of the specialized high-MCT formula, the family transitioned to a more affordable infant formula with a substantially lower MCT content (approximately 33%). Consequently, during subsequent follow-up, the classic clinical features of CMRD became progressively apparent.At the follow-up visit at 5 months of age, the infant's weight was 4.25 kg (severely below the 3rd percentile), length was 59.3 cm, and head circumference was 40 cm. He appeared markedly wasted. Persistent steatorrhea was now a prominent feature. Biochemical analysis showed persistently elevated liver enzymes (ALT 272 U/L, AST 242 U/L). Neurologically, he demonstrated significant developmental delay: head lag on pull-to-sit, inability to maintain head control in the vertical position, and decreased muscle tone in the upper extremities, although visual and auditory tracking were present. By the telephone follow-up at 9 months of age, the family had discontinued clinical visits. The reported weight was only 4.5 kg, indicating virtually no weight gain over the preceding four months.This trajectory demonstrates that while the diagnosis was established early, suboptimal dietary management due to socioeconomic constraints was associated with a failure to achieve catch-up growth, persistence of hepatic dysfunction, and the emergence of severe neurodevelopmental delay. A timeline of key diagnostic and therapeutic events is summarized in Figure 1.

Assessment of Caloric Intake: During the early hospitalization phase, prior to the initiation of the MCT-enriched formula, the infant was fed with a standard infant formula providing approximately 105 kcal/kg/day (equivalent to approximately 160 mL/kg/day), which is considered sufficient for age, yet weight gain remained absent. This observation argues against simple caloric insufficiency as the primary cause of the failure to thrive.

Fat-soluble Vitamin Levels: 25-Hydroxyvitamin D level was measured at 56.1 ng/mL at 5 months of age, which is within the sufficient range. Levels of vitamins A, E, and K were not assessed due to local laboratory limitations.

## 3 Discussion

CMRD is a rare autosomal recessive disorder characterized by defective chylomicron secretion owing to *SAR1B* variants, leading to marked fat malabsorption, hypocholesterolemia, and failure to thrive in infancy (10, 11). Clinical reports consistently describe affected infants presenting with persistent gastrointestinal symptoms, most notably diarrhea, steatorrhea, and vomiting, alongside growth faltering, as observed in cases where diagnosis was delayed until persistent abdominal distension and malnutrition became apparent (12, 13). Laboratory findings typically include low levels of apolipoprotein B, LDL, HDL, and total cholesterol, with marked deficiencies in fat-soluble vitamins (12, 14). Although most CMRD cases in the literature report onset within the first year of life, the present case is notable for its onset in the neonatal period with a failure to gain weight as the singular and initially isolated presenting symptom. The absence of persistent diarrhea or steatorrhea at the initial presentation further complicated early diagnosis. Notably, typical steatorrhea emerged later in the course, underscoring the evolving and sometimes delayed presentation of malabsorption in CMRD. This evolving phenotype suggests that the functional impairment in fat absorption may become more pronounced as dietary fat intake increases or with intestinal maturation, a hypothesis that warrants further investigation.

Differential diagnoses for infants presenting with growth failure and hepatic dysfunction often include urea cycle disorders and fatty acid oxidation defects, both of which may present with nonspecific symptoms such as vomiting, lethargy, and abnormal liver enzymes (15, 16). In these metabolic disorders, tandem mass spectrometry and urine organic acid analysis can reveal overlapping biochemical abnormalities, potentially leading to diagnostic uncertainty, as illustrated by the initial metabolic screening in this case. Furthermore, birth asphyxia and its sequelae, namely hypoxic-ischemic encephalopathy, can contribute to hepatic and neurological dysfunction in neonates; however, they typically present with clear preceding perinatal events and may be distinguished based on neuroimaging and clinical evolution (17, 18). In our patient, the persistence of significantly elevated transaminases (ALT/AST >240 U/L) up to 5 months of age, in the absence of MRI findings characteristic of hypoxic-ischemic injury, makes isolated perinatal asphyxia an unlikely sole explanation for the hepatic involvement. In this case, a definitive diagnosis was established by identifying compound heterozygous likely pathogenic variants in *SAR1B*, a finding that includes the novel c.258G > A (p.Trp86Ter) variant, expanding the variant spectrum of the disease. This genotype has been rarely reported in the literature, particularly in the context of neonatal onset and the absence of classic gastrointestinal symptoms (11, 13). The presence of two truncating variants likely results in complete loss of protein function, which may correlate with the early and severe biochemical phenotype observed, including profound hypocholesterolemia and hepatic dysfunction. Therefore, this study expands the recognized phenotypic and genotypic spectrum of CMRD, highlights the diagnostic pitfalls posed by biochemical overlap with other metabolic diseases, and underscores the critical role of genetic testing in resolving challenging early infantile presentations.

The distinction between inadequate intake and primary malabsorption is critical. In this case, several lines of evidence argue strongly against simple undernutrition as the sole cause. Most notably, during the initial hospitalization and before the diagnosis was established, the infant received intensified feeding with a standard formula providing approximately 105 kcal/kg/day (equivalent to approximately 160 mL/kg/day). The persistence of poor weight gain despite this calorically sufficient intake is inconsistent with a diagnosis of isolated caloric deficiency. Instead, it underscores an inherent defect in nutrient utilization. This observation, combined with the profound hypocholesterolemia, coagulopathy, and elevated transaminases—biochemical markers not typically prominent in pure starvation—collectively points to a primary metabolic and absorptive disorder. The subsequent dramatic clinical and biochemical improvement only after switching to a low-fat, MCT-enriched formula provides further, confirmatory evidence that the core pathophysiology was a defect in long-chain fatty acid absorption and chylomicron assembly, consistent with CMRD. This case also provides several instructive teaching points, particularly regarding the diagnostic pitfalls and importance of a systematic approach for evaluating infants with failure to thrive and hepatic dysfunction. The nonspecific and variable presentation of CMRD, which may mimic more common etiologies, such as nutritional insufficiency, gastrointestinal diseases, or inborn errors of metabolism, including urea cycle defects and fatty acid oxidation disorders, presents a major diagnostic challenge. Notably, metabolic screening in our patient revealed abnormalities suggestive of urea cycle and fatty acid oxidation defects, both of which frequently present with hepatic dysfunction, vomiting, and growth failure during infancy, thereby increasing the risk of misdiagnosis (15). Furthermore, the lack of overt steatorrhea or persistent diarrhea during the neonatal period, as observed in this case, underscores the need for heightened clinical suspicion and supports the notion that the phenotypic spectrum of CMRD is broader than previously understood (3). Endoscopic and histopathological findings, when available, may reveal lipid-laden enterocytes; however, these are neither universally present nor specific to CMRD, underscoring the limitations of traditional diagnostic modalities (3, 7).

The advent and integration of next-generation sequencing has revolutionized the diagnostic workflow for rare metabolic diseases and is especially valuable when standard biochemical and clinical findings are inconclusive (8). In cases of unexplained hypocholesterolemia with fat-soluble vitamin deficiency, prompt genetic analysis of *SAR1B* is crucial, as timely identification of pathogenic variants facilitates early dietary intervention, which is crucial for improving growth and preventing neurological and hepatic complications (6). Furthermore, given the autosomal recessive inheritance and possibility of recurrence in siblings, genetic counseling for affected families is essential. Regarding its management, evidence supports the use of a low-fat diet enriched with MCT along with supplementation with fat-soluble vitamins, which bypasses the defective chylomicron transport pathway, supports catch-up growth, and mitigates hepatic injury in patients with CMRD (6, 19). However, our case starkly illustrates that the efficacy of this therapy is entirely dependent on strict and sustained adherence. The initial clinical and biochemical improvements were reversed when dietary compliance waned, leading to the emergence of classic steatorrhea, arrested growth, and neurodevelopmental delay. This underscores that CMRD management is a lifelong commitment, and socioeconomic barriers to treatment adherence must be addressed as part of comprehensive care. This case highlights the indispensable role of comprehensive genetic and metabolic evaluation in infants with atypical presentations of malnutrition and liver dysfunction and highlights the necessity of a multidisciplinary approach to optimize outcomes.

Reflecting on this case, it is evident that early recognition of rare inherited metabolic disorders such as CMRD is critical, particularly in neonates or infants primarily presenting with a failure to thrive and subtle hepatic abnormalities. The absence of prominent gastrointestinal symptoms and the predominance of nonspecific findings during the early stages of the disease, as observed in this patient, pose significant challenges for clinicians and increase the risk of delayed or missed diagnoses. This case highlights the need for clinicians to maintain a high degree of clinical suspicion for rare lipid malabsorption syndromes in the differential diagnosis of unexplained growth faltering, especially when accompanied by hypocholesterolemia and fat-soluble vitamin deficiency. Prompt genetic testing, even in the absence of classic features, enables the timely initiation of dietary modification and vitamin supplementation, which can substantially improve outcomes and mitigate the risk of irreversible hepatic or neurodevelopmental sequelae (20).

This case also underscores a sobering reality in the management of chronic, diet-dependent metabolic disorders: an early diagnosis alone is insufficient to guarantee a favorable outcome. Our patient's initial positive response to the correct dietary therapy highlights its potential efficacy. However, the subsequent course, characterized by poor weight gain, persistent hepatic injury, and emergent neurodevelopmental delay, starkly illustrates the consequences of inadequate long-term dietary control. The primary barrier in this case was the prohibitive cost of the specialized, high-MCT formula, leading the family to substitute a less effective alternative. This experience brings to the forefront the often-overlooked socioeconomic challenges in managing rare diseases. It argues compellingly for healthcare systems and clinicians to develop sustainable, affordable nutritional strategies and provide robust psychosocial and economic support to ensure treatment adherence, without which the benefits of early genetic diagnosis are negated.

Furthermore, the evolution of symptoms in our patient—from isolated failure to thrive to classic steatorrhea and then to neurodevelopmental impairment—provides a natural history vignette of untreated or suboptimally treated CMRD. It reinforces that neurological injury is not always a late-presenting feature but can evolve rapidly in infancy if metabolic control is not achieved. Therefore, management must include vigilant and regular monitoring of growth, nutritional biomarkers, and neurodevelopment, with immediate intervention at any sign of deterioration.

Nevertheless, this report and the broader understanding of CMRD have several limitations. First, the lack of long-term follow-up limits the assessment of sustained growth, neurocognitive development, and liver health, as longitudinal outcomes remain poorly reported in the literature (21). Our own follow-up, though extended to 9 months of age, was compromised by incomplete treatment adherence, highlighting a real-world challenge rather than delineating the natural history under optimal care. Second, the precise mechanisms underlying secondary hepatic injury and coagulopathy in CMRD remain unclear, necessitating further investigation into genotype-phenotype correlations and extraintestinal manifestations (5, 20). Third, ancillary diagnostic tests, such as direct measurements of intestinal fat absorption and serial monitoring of apolipoprotein B and vitamins A, E, K, were not performed, reflecting practical constraints and highlighting a common limitation in real-world clinical settings. Considerably future investigations should include long-term assessments of sustained growth and neurodevelopment in patients with CMRD under standardized management, integrating animal models and cell studies to understand and explore the molecular mechanisms of CMRD hepatic injury. Overall, this case reinforces the need to enhance the recognition and diagnosis of rare lipid metabolic disorders among healthcare professionals, develop multicenter collaborations for data sharing, and advance research that elucidates the full clinical spectrum and optimizes the management of CMRD and related conditions (20).

## 4 Patient’s perspective

From the patient's perspective, the patient's parents shared that reading this case report provided them with a deeper understanding of their son's condition. They expressed that the report helped clarify the clinical and genetic aspects of the diagnosis, allowing them to better grasp the implications for their son's health and future care.

## Data Availability

The datasets presented in this study can be found in online repositories. The names of the repository/repositories and accession number(s) can be found in the article/Supplementary Material.
