# Supplementary material for: Case Report: Neonatal-onset chylomicron retention disease presenting as isolated failure to thrive with compound heterozygous SAR1B variants: the value of early genetic testing and challenges of long-term management
Source: Front Pediatr. 2026 Feb 12;14:1684900. doi: 10.3389/fped.2026.1684900 (PMC12935971; doi:10.3389/fped.2026.1684900)
Supplement: Supplementary file 1 [file Supplementaryfile1.docx]

**English Description of Genetic Testing Report**

File Name: DDN25013314_NTb01F.pdf

1. Overview

This is a genetic testing analysis report in Chinese, issued by a Chinese laboratory. It details the results of trio whole-exome sequencing (WES) performed on a proband (a 2-month-19-day-old male infant) and his parents. The testing was conducted to investigate the genetic etiology of the proband's clinical presentation, which included liver injury, failure to thrive, and prolonged neonatal jaundice.

2. Key Findings

The report identified pathogenic variants highly relevant to the proband's phenotype in the SAR1B gene:

Variant 1: c.442C>T (p.Arg148Ter). Heterozygous, inherited from the father. ACMG classification: Likely Pathogenic.

Variant 2: c.258G>A (p.Trp86Ter). Heterozygous, inherited from the mother. ACMG classification: Likely Pathogenic.

Inheritance Pattern: Compound heterozygous. The proband inherited two different likely pathogenic variants from his parents.

Associated Disease: Chylomicron Retention Disease (CMRD; OMIM: 246700), an autosomal recessive disorder characterized by severe fat malabsorption and failure to thrive in infancy.

Conclusion: The genetic findings support a molecular diagnosis of Chylomicron Retention Disease (CMRD) caused by compound heterozygous likely pathogenic variants in the SAR1B gene, explaining the proband's clinical features (liver injury, growth delay).

3. Other Findings / Variants of Uncertain Significance (VUS)

The report lists two variants of uncertain significance with unclear or weak relevance to the primary phenotype:

UGT1A1 gene, Variant: c.1091C>T (p.Pro364Leu), Heterozygous. This variant is associated with disorders of bilirubin metabolism (e.g., Gilbert syndrome, Crigler-Najjar syndrome). However, the proband is only a heterozygous carrier, and his primary phenotype does not align with these conditions.

SLC25A15 gene, Variant: c.398G>A (p.Arg133Gln), Heterozygous. This variant is associated with HHH syndrome (Hyperornithinemia-Hyperammonemia-Homocitrullinuria syndrome). Again, the proband is a heterozygous carrier, and his phenotype is not a typical match.

4. Technical Details & Quality Control

Methodology: Trio Whole-Exome Sequencing (WES) using IDT capture probes.

Data Quality: Excellent sequencing metrics were achieved for all three samples. Target region coverage was >99.6%, >20X coverage was >98.7%, >50X coverage was >97.2%, and Q30 scores were >97%.

CNV/UPD Analysis: Within the technical scope and reporting thresholds of this WES assay, no pathogenic copy number variations (CNVs) or uniparental disomy (UPD) explaining the phenotype were detected.

Limitations: The report includes a comprehensive list of technical limitations, noting that WES has inherent constraints in detecting large CNVs, dynamic mutations, certain structural variants, and non-coding variants. All results should be interpreted in the context of the clinical phenotype, and orthogonal confirmation may be recommended.

5. Clinical Utility

This report provides a clear molecular diagnosis, linking the patient's symptoms to Chylomicron Retention Disease (CMRD) due to SAR1B mutations. This information is crucial for:

Confirming Diagnosis: Establishing the genetic cause and explaining the clinical manifestations.

Guiding Management: Informing treatment strategies as per CMRD guidelines, including supplementation of fat-soluble vitamins (especially vitamin E), a diet low in long-chain fats, and regular nutritional monitoring.

Genetic Counseling: Clarifying the autosomal recessive inheritance pattern for accurate family recurrence risk assessment.

Differential Diagnosis: The comprehensive exome analysis significantly reduces the likelihood of other monogenic disorders being responsible for the current phenotype.

Note: This is an English summary of the key contents of the original Chinese report. All clinical decisions should be based on a complete clinical evaluation and the original report, under the guidance of qualified healthcare professionals.
